# Molecular cytogenetic characterization of *Dasypyrum breviaristatum* chromosomes in wheat background revealing the genomic divergence between *Dasypyrum* species

**DOI:** 10.1186/s13039-016-0217-0

**Published:** 2016-01-25

**Authors:** Guangrong Li, Dan Gao, Hongjun Zhang, Jianbo Li, Hongjin Wang, Shixiao La, jiwei Ma, Zujun Yang

**Affiliations:** School of Life Science and Technology, University of Electronic Science and Technology of China, Chengdu, 610054 China

**Keywords:** *Dasypyrum breviaristatum*, Fluorescence *in situ* hybridization, Molecular marker, Wheat

## Abstract

**Background:**

The uncultivated species *Dasypyrum breviaristatum* carries novel diseases resistance and agronomically important genes of potential use for wheat improvement. The development of new wheat-*D. breviaristatum* derivatives lines with disease resistance provides an opportunity for the identification and localization of resistance genes on specific *Dasypyrum* chromosomes. The comparison of wheat-*D. breviaristatum* derivatives to the wheat-*D. villosum* derivatives enables to reveal the genomic divergence between *D. breviaristatum* and *D. villosum*.

**Results:**

The mitotic metaphase of the wheat- *D. breviaristatum* partial amphiploid TDH-2 and durum wheat -*D. villosum* amphiploid TDV-1 were studied using multicolor fluorescent *in situ* hybridization (FISH). We found that the distribution of FISH signals of telomeric, subtelomeric and centromeric regions on the *D. breviaristatum* chromosomes was different from those of *D. villosum* chromosomes by the probes of Oligo-pSc119.2, Oligo-pTa535, Oligo-(GAA)_7_ and Oligo-pHv62-1. A wheat line D2139, selected from a cross between wheat lines MY11 and TDH-2, was characterized by FISH and PCR-based molecular markers. FISH analysis demonstrated that D2139 contained 44 chromosomes including a pair of *D. breviaristatum* chromosomes which had originated from the partial amphiploid TDH-2. Molecular markers confirmed that the introduced *D. breviaristatum* chromosomes belonged to homoeologous group 7, indicating that D2139 was a 7V^b^ disomic addition line. The D2139 displayed high resistance to wheat stripe rust races at adult stage plant, which may be inherited from, *D. breviaristatum* chromosome 7V^b^.

**Conclusion:**

The study present here revealed that the large divergence between *D. breviaristatum* and *D. villosum* with respected to the organization of different repetitive sequences. The identified wheat- *D. breviaristatum* chromosome addition line D2139 will be used to produce agronomically desirable germplasm for wheat breeding.

## Background

The genus *Dasypyrum* (or *Haynaldia*) consists of only two diploid species, the annual *Dasypyrum villosum* and perennial *D. breviaristatum* [1]*.* The genomes of diploid *D. villosum* and *D. breviaristatum* were assigned the symbols V and V^b^, respectively [2, 3]. Based on the sequences comparison of nr5S DNA multigene family, Baum et al. [4] suggested that the genome constitution of 4x *D. breviaristatum* should be considered as an allotetraploid VVV^b^V^b^. *Dasypyrum* species possess agronomically important genes such as disease resistance, high protein quality and drought tolerance, all of which represent valuable resources for global wheat breeding [3]. The species *D. villosum* has been extensively hybridized with wheat for at least 6 decades, and several disease resistance genes have been successfully transferred to wheat [5–7]. Above all, over 20 elite cultivars carrying the wheat- *D. villosum* chromosome T6AL · 6VS translocation with powdery mildew resistance gene *Pm21* have been released into agricultural production in China [8, 9]. Given the widespread success of this introgression from *D. villosum*, researches have been conducted with a similar aim to transfer useful genes from *D. breviaristatum* into wheat. Subsequently, the wheat- *D. breviaristatum* partial amphiploid [10] and wheat- *D. breviaristatum* introgression lines with multiply disease resistances have developed [11, 12].

Molecular and cytogenetic methods have been previously employed to assess the level of chromosomal divergence of these two *Dasypyrum* species [13, 14]. A large number of interspecific and intraspecific chromosome variations and significant genomic diversification were observed among different *Dasypyrum* accessions, probably due to the out-crossing characteristic of these two *Dasypyrum* species [2]. Each pair of *Dasypyrum* chromosomes were transferred into a wheat background after intergeneric hybridizations. The wheat- *Dasypyrum* chromosome addition lines with different *Dasypyrum* species or accession origins allow comparison of the different *Dasypyrum* genomes in wheat backgrounds. In the present study, fluorescent *in situ* hybridization (FISH) was carried out to characterize differences between *D. breviaristatum* and *D. villosum* chromosomes by comparing karyotypes between the wheat- *D. breviaristatum* partial amphiploid TDH-2 and *Triticum turgidum* - *D. villosum* amphiploid TDV-1, The FISH and molecular markers were applied to identify new wheat- *D. breviaristatum* addition line with stripe rust resistance, which will be a useful germplasm for wheat genetics and breeding.

## Results

### Comparative FISH karyotype of TDH-2 and TDV-1

**Fig. 1:**
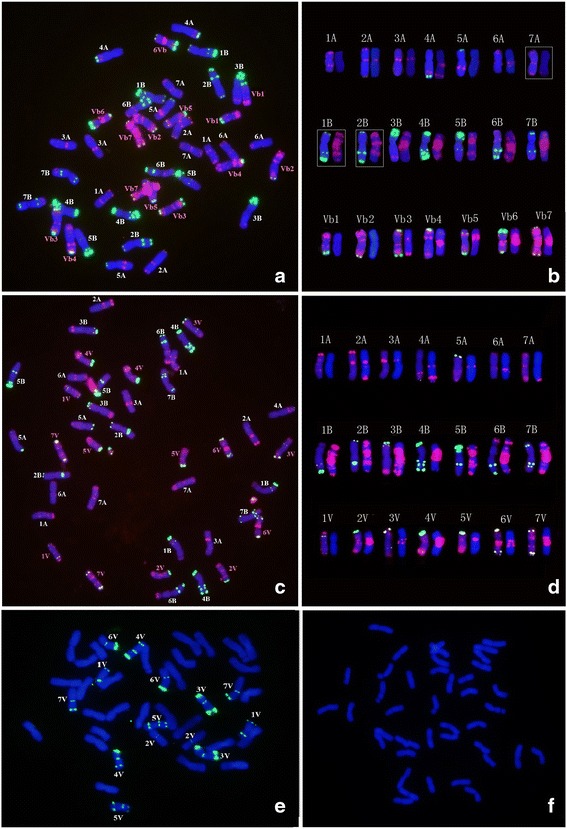
FISH and karyotypes of TDH-2 (**a**, **b**, **f**) and TDV-1(**c**, **d**, **e**). The mitotic metaphase chromosomes (**a**, **c**) after hybridization with probes Oligo-pTa535 (red) and Oligo-pSc119.2 (green), or (**b**, **d**) hybridized with Oligo-(GAA)_7_ (red) The boxes shows the modified chromosomes compared to its parents. Figures **e** and **f** are hybridized by probe of pHv62-3 (green)

The mitotic metaphase chromosomes of the wheat- *D. breviaristatum* partial amphiploid TDH-2, were hybridized with probes Oligo-pSc119.2, Oligo-pTa535, Oligo-(GAA)_7_ by sequential multicolor-FISH (Fig. 1). As shown in Fig. 1a, the FISH hybridization signals of the probes Oligo-pSc119.2 and Oligo-pTa535 can easily identify the 28 wheat chromosomes from 1A-7A and 1B-7B based on the standard FISH karyotype of wheat chromosomes using the same probes described by Tang et al. [15]. Yang et al. [10] reported that the partial amphiploid TDH-2 was produced by the elimination of some chromosomes from the wheat Chinese Spring (CS)- *D. breviaristatum* decaploid amphiploid. It is likely that the A and B chromosomes of TDH-2 originated from CS. By comparing the FISH patterns of Oligo-pSc119.2 and Oligo-pTa535 probes of TDH-2 to those of CS by Tang et al. [15], we found additional signals corresponding to probe Oligo-pSc119.2 on the terminal regions of 1BS and 2BL in TDH-2 (Fig. 1b). After comparing the (GAA)n signal distribution on TDH-2 chromosomes with those of CS reported by Danilova et al. [16], we observed that the (GAA)n signals on chromosome 7A of CS were absent in TDH-2. The results suggest that at least three wheat chromosomes have undergone structural change(s) which may be related to the presence of *D. breviaristatum* chromosomes. Each of the seven pairs of *D. breviaristatum* chromosomes were also distinguished using probes Oligo-pSc119.2, Oligo-pTa535 in TDH-2 (Fig. 1b). These chromosomes were temporarily named Vb1-Vb7 (Fig. 1b).

FISH using probes Oligo-pSc119.2, Oligo-pTa535, Oligo-(GAA)_7_ were also carried out on the chromosomes of the *Triticum turgidum* cv. Jorc-69- *D. villosum* amphiploid TDV-1 (Fig. 1c and d). We found that the signals of probe Oligo-pSc119.2 were mainly located on terminal sites, while the hybridization signals of Oligo-pTa535 were distributed along the chromosome arms of *D. villosum*. The probe Oligo- (GAA)_7_ hybridized to 2V-7V of *D. villosum* chromosomes at their centromeric or sub-terminal regions. Moreover, we produced a high tandem repeat sequences probe Oligo-pHv62-1 as reported by Li et al. [17]. FISH revealed that Oligo-pHv62-1 present in terminal or sub-terminal heterochromatic C-banding regions of *D. villosum* chromosomes in TDV-1, but was absent in *D. breviaristatum* chromosomes of TDH-2 (Fig. 1e and f). The comparative FISH karyotypes of the *D. breviaristatum* and *D. villosum* chromosomes allows easily to distinguish each individual *Dasypyrum* chromosome in wheat background.

### FISH of wheat- D. breviaristatum addition line D2139

**Fig. 2:**
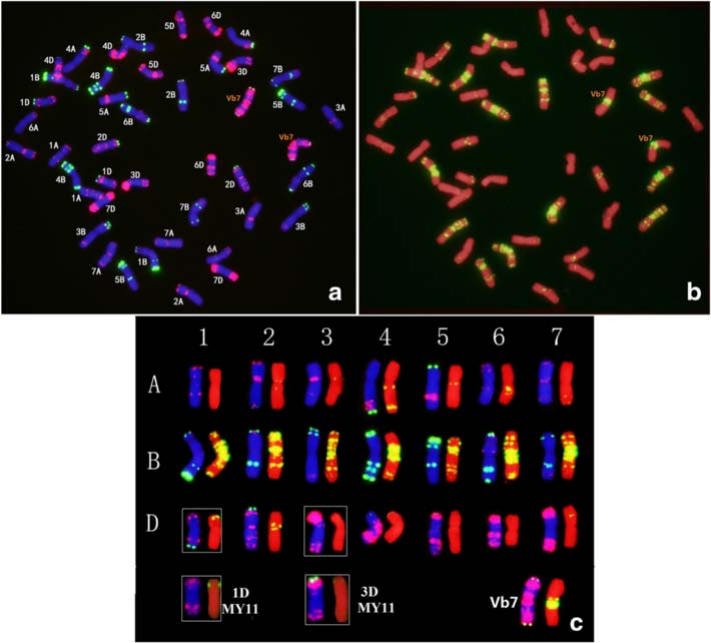
Sequential FISH karyotypes of D2139. Left figure (**a**) was stained with DAPI (blue), Oligo-pTa535 (red) and Oligo-pSc119.2 (green), while the right figure (**b**) chromosomes were stained by PI (red) and Oligo-GAA (green). The box shows the modified chromosomes compared to the parent line MY11 (**c**)

Sequential multi-color ND-FISH by probes Oligo-pSc119.2, Oligo-pTa535, Oligo-(GAA)_7_ was conducted to characterize the mitotic metaphase cells of D2139 (Fig. 2). The chromosome number of D2139 is 2n = 44, including all the 42 wheat chromosomes and two alien chromosomes added in the wheat background. The probes Oligo-pSc119.2, Oligo-pTa535, showed a pair of chromosomes with faint Oligo-pSc119.2 hybridization signals at the telomeric region of long arm, and strong hybridization signals of Oligo-pTa535 along the long and short arm in D2139 (Fig. 2). The FISH hybridization pattern of the chromosomes was identical to *D. breviaristatum* chromosomes Vb7 (Fig. 1). Therefore, we concluded that the line D2139 was a chromosome Vb7 addition line. Comparing the FISH patterns of D2139 parents MY11 [15] and TDH-2 (Fig. 1), it appeared that the D2139 line inherited the A and B- genome chromosomes from MY11 and/or TDH-2. Based on the FISH patterns, D2139 inherited chromosomes 5A, 7A, 1B, and 7B which were identical to the TDH-2 parent, while 6B, 2B appeared to be from MY11. Since there is no D-genome in the partial amphiploid TDH-2, D2139 would have inherited D-chromosomes from MY11. As shown in Fig. 2c, chromosomes 1D and 3D revealed clear differences in the distribution of Oligo-pSc119.2 signals compared to previously published FISH patterns of D-genome chromosomes of MY11 [15]. The terminal region of 1DL showed strong Oligo-pSc119.2 signals, while the Oligo-pSc119.2 signals were absent from the 3DS terminal region. The observation implies that the transmission of the *D. breviaristatum* chromosomes may be associated with structural changes in wheat chromosomes.

### Molecular markers analysis

**Fig. 3:**
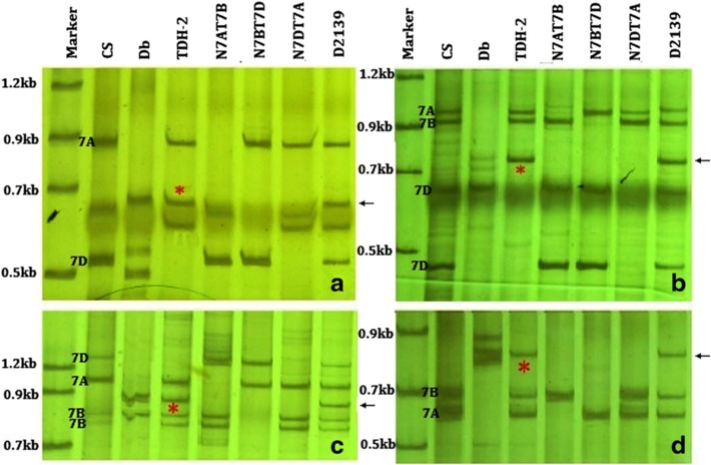
PCR using PLUG primers TNAC1782/*Hae*III (**a**), TNAC1811/TaqI (**b**), TNAC1812/*Taq*I (**c**) and TNAC1867 (**d**). The stars and arrows indicate the *D. breviaristatum* specific bands in TDH-2 and D2139, respectively

PLUG primers were designed from rice genomic DNA sequences specific for the syntenic regions, in the expectation that they would presumably amplify fragments from the corresponding linkage group(s) of wheat genomes [18]. Our previous studies showed that the PLUG markers were useful for producing alien chromosome-specific markers [19, 20]. A total of 21 PLUG markers from wheat homoeologous group 7 were tested on D2139 compared to its parents MY11 and TDH-2 (Table 1). Based on the amplification of nulli-tetrasomic lines of Chinese Spring, the PLUG markers give rise to the 7A, 7B and 7D specific bands, respectively (Fig. 3). A total of 13 pairs of primers generated the identical bands from *D. breviaristatum* V^b^V^b^ and TDH-2 to those from the disomic addition line D2139. These results suggested that the *D. breviaristatum* chromosome in D2139 belonged to homoeologous group 7. Five markers out of the 13 had previously been mapped onto the short arms and eight mapped onto the long arms of group 7 (Table 1). After combining these PCR results with the FISH patterns, we concluded that the D2139 was a 7V^b^ addition line, and the Vb7 was chromosome 7V^b^. The wheat CS - *D. villosum* 7V addition line TA7683 was also used to test the PLUG markers. We found that seven of 13 primer pairs showed polymorphic amplification differences between the *D. breviaristatum* 7V^b^ and *D. villosum* 7V chromosomes (Table 1). The results suggested that sequence divergence may have occurred among *Dasypyrum* species during their evolution.

**Table 1:** The primers used to localize the Vb7 specific amplification in D2139

No.	Primers name	Primer sequences (5’–3’)	Wheat bin map	Restriction enzyme	Length of Vb7 bands
1	TNAC1776	F: ATCATCCTGCTGCTACTGTGC	7AS2-0.73–0.83	*–*	
		R: CCTTCTCAGCTTAGCGATGTG	4AL2-0.75–0.80		
			7DS4-0.73–1.00		
2	TNAC1782	F: TCACTGAACAGCCTAGACATGG	7AS2-0.73–0.83	*Hae*III	690 bp
		R: ATTCGCAGACCGCATCTATC	7BS2-0.27–1.00		
			7DS4-0.73–1.00		
3	TNAC1803	F: TGCGACCAGTCTCTTTGAAAT	C-7AL1-0.39	*Hae*III	800 bp*
		R: GTCGGAGCCTGGATCTCTAGT	7BL2-0.38–0.63		
			7DL5-0.30–0.61		
4	TNAC1806	F: ATTCCTCGTGAATTGCTGGAT	7AS8-0.45–0.59	*Taq*I	350 bp*
		R: TCTGCAGTTAGGGACTTGAAA	7BS2-0.27–1.00		
			7DS2-0.61–0.73		
5	TNAC1811	F: CTGCTCAACGAGTTCATCGAC	7AL1-0.39–0.63	*Taq*I	740 bp
		R: TTGGAGTGGACGTTGCATT	7BL2-0.38–0.63		
			7DL5-0.30–0.61		
6	TNAC1812	F: ACTTCGCTTGGTCTCCTCAAT	7AL5-0.63–0.71	*Taq*I	860 bp
		R: GAGAAGTGTGCCAATTCCAAA	7BL7-0.63–0.78		
			7DL5-0.30–0.61		
7	TNAC1815	F: AGCAGACATCAGCAAGTTTGAG	7AL1-0.39–0.63	*Taq*I	600 bp*
		R: ACTGACAAGCCCATGATTGAC	7BL2-0.38–0.63		
			7DL5-0.30–0.61		
8	TNAC1822	F: CCCTCCGTCCGTGCAAAT	7AL5-0.63–0.71	*Taq*I	730 bp*
		R: GGCTGATGATGGAGACGTG	7BL2-0.38–0.63		
			7DL2-0.61–0.82		
9	TNAC1867	F: GCCTTTCCTTTGGTAGTCTGG	C-7AL1-0.39	*–*	840 bp
		R: CGATCCAAATGATCCTGAAGA	7BL2-0.38–0.63		
			7DL1-0.14–0.30		
10	TNAC1903	F: TCGCTTCTTCTGCTTGTTCTT	C-7AL1–0.39	*Taq*I	920 bp*
		R: CTGCTACTAGGCCACCCAAA	C-7BL2–0.38		
			7DL1-0.14–0.30		
11	TNAC1926	F: CGTCAGCTACAGCGACATCTA	C-7AS8–0.45	*Taq*I	700 bp*
		R: AACTTGAGCAGCGTGGTGTT	7BS2-0.27–1.00		
			7DS3-0.15–0.36		
12	TNAC1943	F: GCTGCTATGGTCCACGAATTA	7AS5-0.59–0.73	*Hae*III	600 bp*
		R: AGAGTATCGTATCCGGGCAAT	7BS2-0.27–1.00		
			7DS4-0.73–1.00		
13	TNAC1957	F: TCAACATTTGCAGGATTGTCA	7AL21-0.74–0.86	*–*	730 bp
		R: TTTCACAGGAACCTCTGCATC	7BL10-0.78–0.84		
			7DL2-0.61–0.82		

The primers and the location in wheat bin map were referred to Ishikawa et al. [18]. The star indicated the Vb7 specific bands were polymorphic to 7V band

### Agronomic traits and rust resistance analysis

**Fig. 4:**
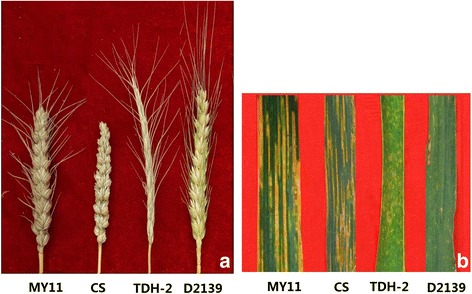
The spikes morphology (**a**) and leaf response to stripe rust (**b**) of the lines

The spike phenotype and stripe rust resistance of lines D2139, TDH-2, CS and MY11 were observed. As shown in Fig. 4a, the spike length of D2139 was 11–12 cm, which was longer than the wheat parents (9–10 cm), while the D2139 had 22–22 spikelets per spike, closely resembling MY11. When inoculated with *P. striiformis* f. sp. *tritici* (PST) races CYR32 and CYR33 at adult plant stage, the TDH-2 and D2139 lines were highly resistance to the isolates, whereas wheat MY11 and CS were highly susceptible (Fig. 4b). These results indicated that the stripe rust resistance in D2139 was from the TDH-2 parent, and originates from *D. breviaristatum*.

## Discussion

With respect to the genomic relationship between two *Dasypyrum* species, *D. breviaristatum* and *D. villosum*, cytogenetic and molecular evidence has revealed the huge genetic divergence between the two species. Friebe et al. [21] established the C-banded patterns of *D. villosum*, and then Linde-Laursen and Frederiksen [22] observed extensive C-band karyotype differences between the two genomes. De Pace et al. [23] isolated a repeat sequence that was mapped further distally on *D. villosum* chromosomes using FISH compared to wheat chromosomes. Galasso et al. [13] found that the differentiated GISH patterns reflected the large genomic divergence between the *D. villosum* and *D. breviaristatum* chromosomes. Liu et al. [14] used FISH probed by a ribosomal DNA sequence and proposed a hypothesis that the diploid *D. villosum* and tetraploid *D. breviaristatum* evolved in parallel from an ancestral species. Recently, we used the pDbC2 probe to hybridize to tetraploid *D. breviaristatum* and diploid *D. villosum* chromosomes, and found more Ty3-gypsy retrotransposon copy numbers in centromeric regions of *D. villosum* than those in *D. breviaristatum* [24]. In the present study, we compared *D. breviaristatum* and diploid *D. villosum* chromosomes through the distribution of FISH signals of Oligo-pSc119.2, Oligo-pTa535, Oligo-(GAA)_7_ by sequential multicolor-FISH (Fig. 1) and the molecular markers (Table 1) by PCR. The results suggested that strong evolutionary divergence involving copy number of repeated sequences and nucleotide sequence rearrangement may have occurred among *Dasypyrum* species during species evolution. Moreover, the Oligo-pHv62-1 can hybridize *D. villosum* chromosomes in TDV-1 (Fig. 1e). It confirmed that the high tandem repeat sequences present largely in telomeric heterochromatin regions of *D. villosum* as reported by Li et al. [17]. However, FISH revealed that Oligo-pHv62-1 was absent in *D. breviaristatum* chromosomes of TDH-2 (Fig. 1f). This significant amplification of different types of repetitive sequences between the *D. villosum* and *D. breviaristatum* chromosomes may be related to adaptation of the plant species to their environments [3]. The cytogenetic and molecular markers which are species-specific can be used to identify and characterize the introgression of *D. villosum* and *D. breviaristatum* chromosome segments into a wheat background.

Rapid genomic and epigenomic changes have been commonly found in some newly synthesized wheat-alien amphiploids [25, 26]. Alterations of alien chromosomal structure in wheat background have also been described especially wheat-rye chromosome addition, substitution and translocation lines [27, 28]. However, the variations in the karyotype of wheat chromosomes have been less reported. Recently, Fu et al. [29] reported that pSc119.2 FISH signals could be observed at the telomeric regions of 3DS arms which was not observed in the current material, and structural variation and abnormal mitotic behavior of the 3D chromosome were detected in the selfed progeny of wheat “MY11”-rye 6R monosomic addition line. Furthermore, Fu et al. [30] reported the occurrence of 14 chromosomal rearrangements in wheat -rye hybrids. Our studies found that chromosomes 1B, 2B and 7A of the wheat- *D. breviaristatum* partial amphiploid TDH-2 (Fig. 1), and chromosomes 1D and 3D of the 7V^b^ addition lines D2139 (Fig. 2) showed apparent structural changes revealed by FISH patterns compared to the parental lines. Patokar et al. [31] characterized several novel wheat-*Thinopyrum bessarabicum* recombinant lines carrying intercalary translocations and did not report any observable wheat chromosomal rearrangements using FISH. It is likely that the distant species of genera *Secale* and *Dasypyrum* may induce such structural changes while present in a wheat background, while chromosomes derived from *Thinopyrum* species may not have the same effect due to their close relationship to wheat [32]. Thus, we suggest that the introgression of chromosomes from closely related species may not lead to the significant structural changes of wheat chromosomes, although the introduction of closely related *Aegilops* chromosomes causes massive deletions of wheat chromosomes [33], which were mainly useful for physical mapping of genes. There is the other possibility that the recipient wheat genotype may also increase the chromosomal rearrangement with visible changes of representative repeats. Taking advantage of fast multicolor FISH methods [15], we recently identified some chromosomal changes in high yielding elite cultivars originating from wheat distant hybridization. The association between the visibly rearranged wheat chromosomes and the yield or disease resistances are being verified for breeding purpose.

*D. villosum* chromosomes are known to contain genetic variability of value for incorporation into wheat. At least three sets of *D. villosum* chromosomes addition lines in wheat background have been developed [34–36]. Novel genes including disease resistance and quality-related characters have been found in different wheat- *D. villosum* derived lines [7]. With the aim to transfer novel genes from *D. breviaristatum* to wheat, we identified the two *D. breviaristatum* chromosomes addition lines Y93-1-A6-4 and Y93-1-6-6, which showed novel resistance to powdery mildew isolates and stem rust Ug99 (pathotype TTKSK) [11]. Molecular marker and GISH analysis revealed that those introduced *D. breviaristatum* chromosomes were rearranged chromosomes involved groups 2, 6 and 7. Recently, Li et al. [12] reported a wheat - *D. breviaristatum* substitution line D11-5 possessing a pair of 2V^b^ chromosomes which had replaced wheat 2D. Based on the FISH analysis, we found that the 2V^b^ chromosome in line D11-5 was identical to chromosome Vb2 of TDH-2 (Fig. 1). We thus suggest that chromosome Vb2 can be provisionally assigned to linkage group 2, subject to confirmation using other genetic markers.

In the present study, we identified line D2139 which contained a pair of *D. breviaristatum* chromosomes confirmed herein to be “7V^b^”. This disomic substitution line D2139 may be potentially useful germplasm for agronomic traits including enhance spike length and the stripe rust resistance from the *D. breviaristatum* 7V^b^ into the wheat genome using marker-assisted chromosome engineering [37]. The divergence between the individual *D. villosum* 7V and *D. breviaristatum* 7V^b^ chromosomes was revealed by FISH and molecular markers in wheat background, which will provide the basis for future detailed comparative genomics analysis. Guo et al. [38] compared chromosomes 7el_1_, 7el_2_, 7E(e), and 7E^i^ derived from different *Thinopyrum* species by molecular and cytological methods. In a similar manner, we intend to create hybrid populations between wheat- *Dasypyrum* 7 V and 7V^b^ addition lines for further and direct localization of genes on these alien chromosomes.

## Conclusions

In summary, the different FISH patterns between *D. breviaristatum* and *D. villosum* chromosomes were observed clearly by using different repetitive sequences as probes, which allows to identify the individual *Dasypyrum* chromosomes in wheat background. The changes of FISH patterns of wheat chromosomes were induced by the induction of *D. breviaristatum* to wheat. The *D. breviaristatum* specific molecular markers can be used to assign the homologous group of *D. breviaristatum* to wheat. We identified wheat- *D. breviaristatum* chromosome 7V^b^ addition line with novel stripe rust resistances will be potential useful for wheat breeding. The molecular and cytogenetic markers will assist to trace the *D. breviaristatum* chromatin in wheat background.

## Methods

### Plant materials

*D. breviaristatum* accession PI 546317 was obtained from the National Small Grains Collection at Aberdeen, Idaho, USA. The wheat– *D. breviaristatum* partial amphiploid TDH-2 (genome AABBV^b^V^b^) was as described by Yang et al. [10]. The accession of *Dasypyrum villosum* TA10220 and the Chinese Spring- *D. villosum* chromosome 7 V addition line TA7683 [39] were obtained from Dr. Bernd Friebe of Wheat Genetic and Genomic Resources Center at Kansas State University, Manhattan, KS, USA. The *T. turgidum* cv. Jorc-69- *D. villosum* amphiploid TDV-1 (genome AABBVV) was developed and provided by Prof. Hua-Ren Jiang at Sichuan Agricultural University, China [40]. Line D2139 was obtained from a BC_1_F_5_ generation of the crosses between wheat cultivar ‘Mianyang 11’ (MY11) and TDH-2.

### Fluorescence *in situ* hybridization (FISH)

Seedling root tips were collected and then treated with nitrous oxide followed by enzyme digestion, using the procedure of Han et al. [41]. The synthesized oligo-nucleotide probes Oligo-pSc119.2, Oligo-pTa535, Oligo-(GAA)_7_ were used for identifying the wheat chromosomes according to the description of Tang et al. [15]. A new probe, Oligo-pHv62-1 (5’ CGAAGGATTG AAAAAAGGAA CAATTTCGCA CTTACAGCTC AAAAATATA TGGGACA 3’) was synthesized and labeled at 5’ 6-carboxyfluorescein (FAM) based on high tandem repeat sequences pHv62 in *D. villosum* as reported by Li et al. [17]. The protocol of non-denaturing FISH by the synthesized probes was described by Fu et al. [30]. Photomicrographs of FISH chromosomes were taken with an Olympus BX-51 microscope equipped with a DP-70 CCD camera.

### Molecular marker analysis

DNA was extracted from young leaves of *D. breviaristatum*, TDH-2, TDV-1, lines D11-5 and CS [42]. PCR-based Landmark Unique Gene (PLUG) primers were designed according to Ishikawa et al. [18]. Polymerase chain reaction (PCR) was performed in an Icycler thermalcycler (Bio-RAD Laboratories, Emeryville, CA) in a 25 μl reaction, containing 10 mmol Tris–HCl (pH 8.3), 2.5 mmol MgCl_2_, 200 μmol of each dNTP, 100 ng template DNA, 0.2 U Taq polymerase (Takara, Japan) and 400 nmol of each primer. The cycling parameters were 94 °C for 3 min for denaturation; followed by 35 cycles at 94 °C for 1 min, 55 °C for 1 min, 72 °C for 2 min; and a final extension at 72 °C for 10 min. The amplified products were separated by 8 % PAGE gel as described by Hu et al. [43].

### Disease resistance screening

Wheat- *D. breviaristatum* derivative lines were evaluated for adult-plant resistance to *Pst* strains CYR32 and CYR33 during the 2013 and 2015 cropping seasons as described by Li et al. [12].
